# Supplementary material for: Coverage and Drivers of Vaccinations in Patients with Autoimmune Rheumatic Diseases: An Italian Multicentric Study
Source: Vaccines (Basel). 2025 Dec 6;13(12):1229. doi: 10.3390/vaccines13121229 (PMC12737605; doi:10.3390/vaccines13121229)
Supplement: Supplementary file 1 [file vaccines-13-01229-s001.zip › Suppl files.pdf]

**Supplementary Materials**

Table 1S Multivariate analysis for predictors of vaccine coverage

| Characteristic | OR | 95% CI | p-value |
|---|---|---|---|
| **Diagnosis** | | | |
| RA vs SLE | 0.857 | 0.433, 1.704 | 0.657 |
| **Gender** | | | |
| Males vs Females | 1.245 | 0.629, 2.406 | 0.520 |
| **Age (years)** | 1.008 | 0.988, 1.028 | 0.440 |
| **BMI** | 1.012 | 0.952, 1.078 | 0.708 |
| **Smoking habit** | | | |
| No vs Yes (current or previous) | 1.365 | 0.816, 2.317 | 0.241 |
| **Education level** | | | |
| Primary school vs High school | 1.159 | 0.663, 2.032 | 0.604 |
| Primary school vs University | 1.779 | 0.875, 3.741 | 0.119 |
| **Disease activity** | | | |
| Low vs High | 0.742 | 0.413, 1.351 | 0.322 |
| **Comorbidities** | | | |
| Absent vs Present | 1.361 | 0.819, 2.265 | 0.234 |
| **Immunosuppression level** | | | |
| Mild vs Moderate | 0.798 | 0.317, 2.050 | 0.633 |
| Mild vs High | 0.877 | 0.426, 1.767 | 0.716 |

Multivariable logistic regression for predictors of overall vaccine uptake (having received at least one recommended vaccine). Results are expressed as odds ratios (OR) with 95% confidence intervals (CI). *p*-values were considered significant if <0.05

**Abbreviations**: OR, odds ratio; CI, confidence interval; RA, rheumatoid arthritis; SLE, systemic lupus erythematosus; BMI, body mass index.

Table 2S Post-hoc Chi-square residuals: Age groups by cluster

| Age group | Cluster | StdResid | Sign |
|---|---|---|---|
| 18–49 | Not offered | 0.9374236 | NS |
| | Unaware | -0.3891559 | NS |
| | Skeptical | -0.8133019 | NS |
| 50–64 | Not offered | -3.1675401 | Under-represented |
| | Unaware | 2.7743263 | Over-represented |
| | Skeptical | 1.3557594 | NS |
| ≥65 | Not offered | 2.3982990 | Over-represented |
| | Unaware | -2.4833727 | Under-represented |
| | Skeptical | -0.6612904 | NS |

Post-hoc analysis of standardized residuals from the $\chi^2$ test assessing the distribution of age groups across motivational clusters. Positive residuals indicate over-representation, whereas negative residuals indicate under-representation. We adopted

a standard cutoff of |1.96|. Residuals exceeding this threshold suggest significant over- or under-representation in the corresponding cluster.
**Abbreviation:** StdResid: Standard Residual from $\chi^2$ test; NS, not significant
